# Intensive Care in Patients with Solid Tumours: A Practical Framework for Integrated Onco-Critical Care

**DOI:** 10.3390/cancers18193210

**Published:** 2026-10-04

**Authors:** Alicia Castelo-Loureiro, Amanda Lesmes González de Aledo, Mateo Bover Larroya, Zaira Molina Collado, Cristina Serrano Gómez, Isaías Martín Badía, Jesús González Olmedo, Santiago Terán, Marcos Valiente Fernández, Carlos Heredia-Mena, Joseph Exebio-Jara, Lidia Orejón García, María Carnevali Frías, Carlos Gómez Martín, María Cruz Martín Delgado, Luis Paz-Ares

**Affiliations:** 1 Department of Medical Oncology, Hospital Universitario 12 de Octubre, 28041 Madrid, Spain; mateo.bover@salud.madrid.org (M.B.L.);; 2 Department of Intensive Care Medicine, Hospital Universitario 12 de Octubre, 28041 Madrid, Spain; amanda.lesmes@salud.madrid.org (A.L.G.d.A.); maria.carnevali@salud.madrid.org (M.C.F.);; 3 Department of Internal Medicine, Hospital Universitario 12 de Octubre, 28041 Madrid, Spain; 4 Hospital Ruber Juan Bravo, 28006 Madrid, Spain; 5 Co-Management Working Group, Sociedad Española de Medicina Interna, 28016 Madrid, Spain

**Keywords:** onco-critical care, intensive care unit, solid tumours, critical illness, multidisciplinary care, time-limited ICU trial, frailty, palliative care

## Abstract

The number of patients with solid tumours requiring intensive care is increasing as cancer survival improves and oncological treatments become more complex. Intensive care unit (ICU) admission decisions should not be based on cancer diagnosis, metastatic status or palliative treatment intent alone. This narrative review presents a practical, multidisciplinary framework for assessing critically ill patients with solid tumours. It integrates acute illness severity and reversibility, organ failure, baseline performance status and frailty, cancer trajectory, remaining treatment options, expected functional recovery and patient preferences. We discuss contemporary anticancer treatment toxicities, cancer-associated thromboembolic disease, older adults, palliative care, communication and ethical decision-making. We also propose an algorithm ranging from full ICU support to a time-limited ICU trial with predefined reassessment or comfort-focused care. Early joint assessment by oncology and intensive care teams, followed by dynamic reassessment, may reduce both inappropriate escalation and premature exclusion from potentially beneficial organ support. Future research should prioritize structured pathways, improved prognostic tools and outcomes beyond survival, including functional recovery, quality of life and the ability to resume meaningful cancer treatment.

## Simple Summary

More patients with solid tumours now need intensive care because cancer treatments are more effective and people are living longer after a cancer diagnosis. Cancer stage alone should not determine whether intensive care is offered. Decisions should consider how severe and reversible the acute illness is, the patient’s previous health and independence, the expected course of the cancer, available treatments and the patient’s wishes. This review explains how oncology and intensive care teams can make these decisions together. It also proposes a practical algorithm for full intensive care, a time-limited trial with planned reassessment, or care focused on comfort, depending on the clinical situation.

## 1. Introduction

The number of patients with cancer admitted to intensive care units (ICUs) has increased over recent years. This reflects several changes in clinical practice: patients are older, cancer is more prevalent, major oncological surgery is increasingly complex, and systemic treatments have substantially improved survival. As a result, many patients with solid tumours, including some with advanced disease, may experience acute complications while still having realistic oncological treatment options. In this context, ICU admission should no longer be viewed as exceptional or automatically linked to end-of-life care [1,2,3].

Patients with solid tumours may require ICU admission for very different reasons, including postoperative complications, sepsis, respiratory failure, shock, thromboembolic disease, neurological complications, cancer-related emergencies or treatment-related toxicities. At the same time, an ICU admission may affect subsequent cancer care by worsening performance status, delaying systemic treatment, limiting eligibility for clinical trials or increasing functional dependence after discharge. For this reason, the management of these patients requires early and direct communication between oncologists and intensivists, ideally with the involvement of palliative care, geriatrics, nursing teams, patients and families when appropriate [1,2,3,4].

This review is deliberately focused on adults with solid tumours. It does not aim to provide a comprehensive framework for hematological malignancies, such as leukemias, lymphomas or multiple myeloma, except where syndromes or treatments overlap with solid-tumour care. These conditions have distinct disease trajectories, treatment pathways and ICU-specific complications, and are therefore considered separately from the scope of this review. Some foundational ICU references include mixed cancer populations and are cited only for general principles of critical-care assessment; whenever outcome data are presented as specific to solid tumours, we identify the corresponding solid-tumour cohorts explicitly [5,6,7].

This narrative review provides a practical framework for integrated onco-critical care, focusing on prognostic assessment, ICU admission, communication, toxicities of modern anticancer therapies, thromboembolic disease, older adults, palliative care and ethical decision-making.

## 2. Epidemiology

ICU admission in patients with cancer has become increasingly common, reflecting both the growing number of people living with cancer and the complexity of current oncological care [1,2]. Planned ICU admission after major surgical resections remains frequent and is generally associated with favourable outcomes, particularly when patients have good baseline functional status and admission is part of perioperative management [3,8]. In contrast, unplanned ICU admission due to cancer-related complications, sepsis, acute respiratory failure, shock, urgent surgery or treatment-related toxicity is usually associated with a worse prognosis [3,8].

Despite improvements in cancer care, life-threatening events remain a major challenge for oncologists and intensivists. The prognosis of critically ill patients with cancer has improved over recent decades, probably due to advances in both oncology and intensive care, including better supportive care, earlier recognition of acute deterioration and more appropriate selection of patients who may benefit from ICU support [3,8].

However, the available evidence has important limitations. Many studies have been performed in specialized cancer centres or are based on administrative databases with limited clinical information [3,8]. In addition, older cohorts may not reflect the current prognosis of patients treated with immunotherapy, targeted therapies or other modern systemic treatments [1,2]. Reported outcomes therefore vary widely according to tumour type, disease stage, reason for ICU admission, baseline performance status, frailty, organ failures and need for invasive organ support [3,8].

Recent cohorts focused on solid tumours illustrate why outcomes beyond ICU survival must be considered. In a Spanish cohort, ICU and hospital mortality were 9.4% and 24%, respectively, but functional status deteriorated substantially and anticancer treatment was permanently discontinued in 40% of patients. In a second contemporary cohort of 955 patients, ICU mortality was 23.5%, whereas one-year survival was only 29.3%, underscoring the difference between short-term rescue and longer-term outcome [5,6].

## 3. Population Overview

Patients with solid tumours who require intensive care no longer represent a single prognostic group. In the past, the presence of metastatic disease was often interpreted as a marker of short life expectancy and limited benefit from aggressive supportive care. Although this remains true for some patients, it is no longer an adequate assumption for all. Advances in cancer treatment have created a much more heterogeneous population, ranging from patients with rapidly progressive disease and no further therapeutic options to patients with advanced cancer who may live for years with preserved functional status.

This change is largely explained by a better understanding of tumour biology and the incorporation of molecular and immune biomarkers into routine clinical practice. Modern oncology is increasingly guided not only by tumour site and histology, but also by targetable alterations, immune sensitivity, previous response to treatment and availability of subsequent lines of therapy [9,10]. These factors can substantially modify prognosis and may also influence the expected speed of response to treatment. For this reason, cancer stage alone is often insufficient when estimating whether ICU admission may be appropriate.

Lung cancer illustrates this shift particularly well. Not long ago, metastatic non-small cell lung cancer was usually associated with a median survival of less than one year and few effective systemic options. This picture has changed dramatically in selected molecular subgroups. In patients with ALK-rearranged disease, first-line lorlatinib in the CROWN trial achieved a 5-year progression-free survival rate of approximately 60%; median progression-free survival was still not reached in the lorlatinib arm [11,12]. Immune checkpoint inhibitors have also transformed the prognosis of patients with advanced lung cancer. In KEYNOTE-024, among patients with a PD-L1 tumour proportion score ≥ 50%, pembrolizumab achieved a 5-year overall survival rate of 31.9%, compared with 16.3% with chemotherapy [13,14,15]. Therefore, two patients with stage IV lung cancer may have radically different expected trajectories: one may have rapidly progressive disease with no remaining options, whereas another may have controlled disease, preserved baseline function and several effective treatments still available.

Melanoma provides another practical example of this change. Before effective targeted therapy and immune checkpoint blockade, median survival in advanced metastatic melanoma was measured in months: 7.7 months with temozolomide and 6.4 months with dacarbazine in a phase III trial [16]. The introduction of BRAF/MEK inhibitors and immune checkpoint inhibitors has since transformed outcomes in selected patients [17,18]. In BRAF-mutant metastatic melanoma, dabrafenib plus trametinib achieved a five-year overall survival rate of 34%, with a median overall survival of 25.9 months [17]. Anti-PD-1-based combinations have produced a median overall survival of approximately 72 months in advanced melanoma [18].

These examples illustrate how the natural history of some metastatic solid tumours has changed substantially. However, these advances should not be overinterpreted. Trial populations are selected, and their outcomes cannot be directly extrapolated to critically ill patients, who often have additional prognostic factors such as organ failure, sepsis, frailty, poor performance status, uncontrolled tumour burden or treatment-related complications.

## 4. What Should the Intensivist Know About the Oncology Patient?

A first essential element is the patient’s baseline condition before the acute event. Performance status, functional independence, frailty, comorbidities, nutritional status and cognitive reserve all influence the probability of recovery after critical illness. In oncology, performance status is routinely assessed because it guides treatment decisions, but it can also help estimate the patient’s physiological reserve before ICU admission. A patient with good baseline function and a reversible complication may have a very different prognosis from a patient with poor performance status, progressive disease and no realistic therapeutic options [1,2,19].

A second element is the current oncological trajectory. The term “palliative treatment” should be interpreted carefully. In oncology, it refers to non-curative treatment intent, but it does not necessarily mean end-of-life care or short survival. Some patients receiving palliative systemic therapy may live for years, particularly those with endocrine-sensitive breast cancer, actionable molecular alterations, immunotherapy-sensitive tumours or oligometastatic disease [11,20,21,22,23]. Therefore, intensivists should ask whether the disease is controlled or progressing, whether the patient is responding to treatment, and whether effective therapeutic options remain available.

The reason for ICU admission and its reversibility are equally important. In many patients with cancer, short-term ICU prognosis is driven more by the acute critical illness and the number of failing organs than by the cancer diagnosis itself [1,2,3,19]. Sepsis, acute respiratory failure, shock, pulmonary embolism, postoperative complications, neurological events and treatment-related toxicities should therefore be assessed according to their potential reversibility. ICU admission is more likely to be appropriate when the acute complication is potentially reversible and recovery would allow for continuation of meaningful cancer care.

Treatment-related complications deserve specific attention. Modern anticancer therapies have introduced new ICU scenarios, including severe immune-related adverse events, cytokine release syndrome, tumour lysis syndrome, pneumonitis, myocarditis, neurological toxicity, severe diarrhea or colitis, and complications related to cellular or bispecific therapies [24,25,26]. Some of these complications may be severe but potentially reversible if recognized early and treated appropriately. In these situations, early communication with the oncology team is essential, because management may require treatment-specific interventions such as corticosteroids, immunosuppressive agents, tocilizumab or specific supportive measures.

Another relevant issue is the expected impact of ICU admission on subsequent cancer care. Even when the acute complication is overcome, critical illness may lead to functional decline, delirium, ICU-acquired weakness, prolonged rehabilitation needs or worsening of comorbidities. These consequences may delay systemic therapy, reduce treatment tolerance or make the patient ineligible for further oncological treatment or clinical trials [2,19]. Therefore, ICU benefit should not be understood only as survival to discharge, but also as the possibility of recovering to a level that allows an acceptable quality of life and, when appropriate, continuation of cancer care.

In a 2024 study of 219 patients with solid tumours, 59.6% of ICU survivors resumed anticancer treatment. Deterioration in performance status after intensive care was independently associated with failure to resume treatment and with one-year mortality, supporting functional recovery as a core onco-critical care outcome [7].

Finally, ICU decisions should be dynamic. In patients with uncertain prognosis but potential benefit, a time-limited ICU trial may be appropriate. This means starting full intensive care support with predefined clinical goals and a documented early reassessment plan. Reassessment should usually occur within 48–72 h for rapidly evolving complications such as septic shock, acute respiratory failure or treatment-related toxicity, and may be extended to 3–5 days when the expected trajectory of recovery is slower, provided that the goals, uncertainty and limits of treatment remain explicit. The reassessment should consider the direction of organ failure, vasopressor requirement, respiratory support, control of the precipitating complication and new information about cancer treatment options [2,3,27]. If the patient improves, intensive care may continue with the aim of recovery. If the patient deteriorates despite appropriate support, treatment goals should be reconsidered jointly with oncology, intensive care, palliative care, the patient when possible, and the family.

## 5. What Should the Oncologist Know About Intensive Care Management?

For oncologists, early ICU referral is most relevant when a potentially reversible complication is causing, or is likely to cause, acute organ failure. Common triggers include sepsis, acute respiratory failure, shock, neurological deterioration, acute kidney injury, postoperative complications, pulmonary embolism and severe treatment-related toxicity. Prompt referral may prevent progression to multiple organ failure and allows the oncology and ICU teams to agree early on realistic goals of care [1,2,3,8].

Oncologists should also be familiar with the type and intensity of organ support that may be required in the ICU. This may include close monitoring, high-flow oxygen therapy, non-invasive or invasive mechanical ventilation, vasopressors, renal replacement therapy, management of severe electrolyte disorders, sedation, invasive procedures and infection control measures. These interventions are not neutral: they may be lifesaving, but they can also lead to complications such as delirium, ICU-acquired weakness, infections, prolonged hospitalization, functional decline and the need for rehabilitation after discharge [2,28,29].

The oncologist has a central role in this reassessment. Intensivists need realistic information about the patient’s oncological prognosis, disease trajectory, treatment options, expected response kinetics and baseline function. At the same time, oncologists should understand how critical illness may affect future cancer care. An ICU admission may delay systemic treatment, worsen performance status, reduce tolerance to therapy or make a patient temporarily or permanently ineligible for a clinical trial. Therefore, the expected benefit of ICU care should include not only survival to ICU discharge, but also the likelihood of functional recovery and the possibility of continuing meaningful oncological treatment [2,28,29].

Finally, communication with patients and families should be coordinated early between oncology and intensive care teams. Oncologists often have a long-standing relationship with the patient and can help translate complex oncological information into realistic goals of care. However, inconsistent messages between teams may increase confusion and distress. Whenever possible, discussions about prognosis, treatment intensity, time-limited ICU trials or limitations of life-sustaining therapies should be held early, ideally before clinical deterioration, and should involve the relevant multidisciplinary team, including palliative care when appropriate. These discussions should be shared, consistent and clearly documented, and should help patients and families understand what ICU care can realistically offer, what its potential burdens are, and how decisions can be aligned with the patient’s values and goals [3,4,8,27,28,29].

## 6. What Should the Patient Know About the ICU?

For many patients and families, ICU admission is associated with fear, uncertainty and loss of control. In patients with cancer, it may also be perceived as a sign of poor prognosis or as the beginning of end-of-life care. However, this is not always the case. Some ICU admissions are planned, such as postoperative monitoring after major surgery, while others occur because of acute complications that may be reversible. Information should therefore be individualized according to the clinical situation, cancer prognosis and goals of care.

Patients and families should receive clear information about what ICU care involves. ICU admission usually means closer monitoring, frequent reassessment and intravenous treatments. In some cases, it may also involve invasive procedures or organ support, such as central venous catheters, arterial lines, mechanical ventilation, vasopressors or renal replacement therapy. Explaining these interventions in plain language can help patients and families understand that ICU care is not a single intervention, but a range of possible supports with different aims and burdens [3,28].

Whenever possible, these conversations should take place before an emergency occurs. Discussing preferences about mechanical ventilation, resuscitation, acceptable quality of life, surrogate decision-making and possible treatment limitations should not be interpreted as “giving up”. Rather, it helps ensure that future ICU decisions are medically appropriate and aligned with what matters most to the patient [30,31,32].

Recent randomized evidence suggests that facilitated advance care planning can increase patient engagement and completion of advance directives compared with patient-directed written or web-based information alone, supporting structured conversations before acute deterioration [33].

## 7. Interdisciplinary Team and Integration

An integrated onco-critical care approach can improve the management of patients with solid tumours admitted to the ICU. Joint assessment by oncology and intensive care teams allows earlier recognition of clinical deterioration, more accurate interpretation of cancer prognosis, better identification of reversible complications and more consistent communication with patients and families. This may reduce delayed ICU referral, avoid decisions based on incomplete assumptions and support more appropriate use of full ICU support, time-limited ICU trials or treatment limitation. In a cohort of 780 patients with cancer considered for ICU admission, 22.3% of those initially considered “too well to benefit” were subsequently admitted to ICU after a second request, and this delayed ICU admission was associated with higher 30-day mortality than in those never admitted after the first assessment (45.9% vs. 25.6%) [1].

To support early joint assessment, Table 1 summarizes clinical factors that may influence the expected benefit of ICU admission in patients with solid tumours. These factors should not be interpreted as rigid admission criteria, but as a framework to guide multidisciplinary discussion.

## 8. New Therapies and Clinical Trials

Modern anticancer treatments have created new scenarios for intensive care. Immune checkpoint inhibitors, targeted therapies, bispecific antibodies, antibody–drug conjugates and cellular therapies have improved outcomes in several solid tumours, but they have also introduced toxicity profiles that may be unfamiliar outside oncology. Some of these complications are severe enough to require ICU admission, but they may also be potentially reversible if recognized early and managed appropriately [34,35].

### 8.1. Immune-Mediated Toxicity

Immune checkpoint inhibitors are now widely used in solid tumours, including lung cancer, melanoma, renal cell carcinoma, urothelial cancer and other malignancies. Most immune-related adverse events are mild or moderate, but severe presentations can occur and may involve almost any organ system. ICU admission may be required for pneumonitis, myocarditis, severe colitis, neurological toxicity, adrenal crisis, hepatitis, nephritis or multi-organ immune-mediated toxicity [26,34,35,36].

In a prospective multicentre study of ICU admissions among patients with solid tumours treated with immune checkpoint inhibitors, almost half of admissions were considered likely related to immune-related adverse events, with acute respiratory failure being the most frequent presentation [34]. In another UK series, only a small proportion of acutely admitted patients treated with immune checkpoint inhibitors required ICU care, but immune-mediated toxicities such as pneumonitis and myasthenia gravis accounted for many of these ICU admissions [35]. Neurological toxicities are rare but can be severe, including myasthenia gravis, Guillain–Barré-like syndromes, encephalitis and seizures, sometimes requiring mechanical ventilation, intravenous immunoglobulins, plasma exchange or immunosuppression [37].

A 2025 systematic review identified 169 patients in six ICU population-based studies admitted because of immune-related adverse events. Pneumonitis and neurological toxicities predominated; ICU mortality was 26%, with a further 8% dying during the following three to six months [38]. The available evidence does not support a reliable organ-specific ranking of who is most likely to benefit from ICU admission. Referral should instead be early when a life-threatening toxicity is potentially reversible and organ support is required. Some presentations nevertheless carry particularly high risk: severe neurological immune-related adverse events had 29% hospital mortality in a multicentre ICU series [37], while a systematic review found myocarditis-associated mortality of 37.7% [39]. These figures support urgent ICU evaluation rather than therapeutic restriction. Current NCCN guidance also addresses toxicities caused by immune checkpoint inhibitors, CAR T-cell therapies and T-cell-engaging bispecific antibodies [40].

For ICU teams, the key point is to consider immune-related toxicity in any acutely deteriorating patient receiving or recently exposed to immune checkpoint inhibitors. Early communication with oncology is essential because management may require treatment-specific interventions, including corticosteroids or additional immunosuppression depending on the organ involved and clinical course.

### 8.2. Cell Therapies, Bispecific Antibodies and Cytokine Release Syndrome

Cellular therapies and T-cell engaging treatments are increasingly relevant to solid tumour research and early-phase clinical trials. These therapies may cause cytokine release syndrome (CRS), a systemic inflammatory syndrome that can present with fever, hypotension, hypoxia, capillary leak, coagulopathy and multi-organ dysfunction [26].

Although most experience with CRS comes from hematological malignancies, intensivists and oncologists involved in early-phase trials for solid tumours should be familiar with its recognition and management. Treatment may include supportive care, vasopressors, oxygen or ventilatory support, corticosteroids and anti-IL-6 therapy such as tocilizumab, depending on severity and institutional protocols. CRS should not be interpreted automatically as treatment failure or futility, because many patients can recover if organ support and specific treatment are provided promptly [26].

Solid-tumour experience is now emerging. In a 2024 phase I study of the HLA-G × CD3 bispecific antibody JNJ-78306358, cytokine release syndrome occurred in 48.7% of patients; all events were grade 1–2 and 23.1% received tocilizumab. This illustrates the need for ICU and oncology teams supporting early-phase solid-tumour trials to anticipate protocol-specific monitoring and rapid access to anti-cytokine therapy [41].

### 8.3. Clinical Trial Patients: Special Considerations

Patients enrolled in clinical trials require specific consideration when admitted to the ICU. The general principles of ICU triage are the same as for other patients with cancer: baseline status, reversibility of the acute illness, organ failures, oncological prognosis and patient preferences remain central. However, investigational therapies may have unknown or evolving toxicity profiles, and the trial protocol may include specific recommendations for monitoring, dose interruption, toxicity management, reporting and treatment discontinuation [36].

Patients enrolled in early-phase clinical trials represent a particularly complex subgroup. They often have advanced disease and may have exhausted standard treatment options, but are usually selected for preserved performance status and adequate organ function. ICU admission should therefore not be considered automatically inappropriate. In a retrospective study of 212 patients with advanced cancer referred to a phase I programme, median survival after the first ICU admission was 3.2 weeks. Outcomes differed markedly according to the reason for admission: median survival was 21.5 weeks after a surgical intervention and 2.1 weeks after a non-surgical intervention; patients who underwent cardiopulmonary resuscitation had a median survival of one day. Non-surgical admission, hypoalbuminemia and a higher APACHE II score were independently associated with worse survival [42]. These data support careful selection, early goals-of-care discussions and close coordination between phase I and intensive care teams.

In this setting, the ICU team should be informed as early as possible that the patient is participating in a clinical trial. The oncologist or principal investigator should clarify the investigational drug, mechanism of action, last administration date, expected toxicities, available antidotes or targeted interventions, and whether sponsor- or protocol-specific guidance exists. When needed, the trial team should be contacted to ensure appropriate pharmacovigilance reporting and protocol compliance.

ICU admission should not automatically exclude a patient from ongoing or future clinical trial participation, but the consequences must be anticipated. Critical illness may delay treatment, require dose interruption, worsen performance status or affect eligibility for subsequent therapy. Conversely, in selected patients, ICU support may allow recovery from a reversible toxicity or complication and preserve the possibility of continued oncological benefit. These decisions require individualized assessment and close coordination among oncology, intensive care, research teams and, when appropriate, palliative care.

## 9. Thromboembolic Disease

The incidence of thromboembolic disease in patients with cancer has increased over recent years, mainly due to longer survival, greater exposure to systemic anticancer treatments and improvements in diagnostic techniques. Venous thromboembolism remains one of the leading causes of death in patients with cancer, second only to progression of the underlying malignancy [43].

Pulmonary embolism (PE) is a relevant cause of ICU admission in this population. The denominators should be distinguished: across different ICU series, PE may be a contributing or primary reason for admission in up to 30% of patients with cancer. By contrast, a recent French ICU study enrolled only cancer patients with acute PE; within that selected cohort, PE was the main reason for ICU admission in 77% of cases. Among those patients, 23% were classified as high-risk PE, 9% as intermediate–high-risk and 68% as low-risk [44]. Right ventricular dysfunction has been reported in 10% to 35% of patients with cancer-associated PE, generally with lower rates than those observed in patients without cancer [44,45,46].

Management is particularly challenging in patients with cancer because thrombotic and bleeding risks frequently coexist. In the setting of hemodynamic instability, patients with cancer appear to be less likely to receive reperfusion or advanced supportive strategies than patients without cancer, including systemic thrombolysis, extracorporeal membrane oxygenation and mechanical ventilation. In one series, the use of thrombolysis was 8% in patients with cancer compared with 20.7% in those without cancer, while ECMO was used in 0.7% versus 5.4%, and mechanical ventilation in 5.5% versus 11%, respectively [46].

Anticoagulation remains the cornerstone of treatment, but the optimal strategy must be individualized. No significant differences have been reported regarding the initial use of unfractionated heparin versus low-molecular-weight heparin in this setting, with reported rates of 42% and 39%, respectively. However, patients with cancer have a higher incidence of major bleeding, reported as 25.5% compared with 11.5% in patients without cancer [46]. This bleeding risk is particularly relevant in patients with recent surgery, thrombocytopenia, brain metastases, gastrointestinal or genitourinary tumours, renal impairment or planned invasive procedures.

Despite more conservative treatment strategies, the COMMAND-VTE registry did not show major differences in PE-related mortality within the first month between patients with and without cancer, with rates of 8.3% and 7%, respectively [46]. Similar findings have been reported in other series [44,45,47]. However, all-cause mortality is higher in patients with cancer, with reported rates of 15.9% versus 7.3% [46]. At 90 days, mortality also appears to be increased compared with patients without cancer, with a reported hazard ratio of 1.68 (95% CI, 1.11–2.55), and some series reporting mortality rates as high as 42% [44,45,46,47,48].

These data suggest that PE in patients with cancer should not be managed only as an isolated thromboembolic event. ICU decision-making should integrate the severity of PE, hemodynamic status, right ventricular dysfunction, bleeding risk, cancer trajectory, baseline performance status and availability of further oncological treatment. In high-risk PE, decisions regarding thrombolysis, catheter-directed therapy, surgical embolectomy or ECMO should be individualized and ideally discussed within a multidisciplinary team including intensivists, oncologists, hematologists or thrombosis specialists, and interventional teams when available.

## 10. Older Adults

Older adults represent a growing proportion of patients with solid tumours who may require ICU admission. However, this population is highly heterogeneous, and chronological age alone should not determine decisions about intensive care. Some older adults remain functionally independent, have controlled cancer and may benefit from full ICU support, whereas others have frailty, cognitive impairment, malnutrition, multimorbidity or limited physiological reserve, which may reduce the likelihood of meaningful recovery.

Frailty and baseline function are more informative than age alone. In ICU populations, frailty is consistently associated with higher mortality, functional decline and reduced probability of returning home after discharge [49,50]. Importantly, patients classified as non-frail or mildly vulnerable on the Clinical Frailty Scale (CFS 1–3) have better outcomes than those with higher frailty scores, supporting the concept that “fit” older adults should not be excluded from ICU care solely because of age [50]. In patients with cancer, frailty may also affect tolerance to systemic treatment and the ability to resume oncological therapy after critical illness [51].

A complete geriatric assessment may not be feasible during an emergency, but selected domains (functional status, cognition, nutrition and social support) can provide clinically relevant information [52,53]. These factors help estimate not only survival from ICU, but also the probability of recovering to a level of function and quality of life that the patient would consider acceptable.

Thus, in older adults, frailty and the likelihood of meaningful recovery—not chronological age alone—should shape decisions about the appropriate intensity of ICU support.

## 11. Palliative Care

Palliative care is an essential component of the management of critically ill patients with solid tumours. Its role is not limited to end-of-life care. In the ICU, palliative care can help with symptom control, communication, advance care planning, family support and decision-making when prognosis is uncertain or when the burden of treatment may be high [4,30,54].

The 2024 ASCO guideline recommends early specialist interdisciplinary palliative care alongside active anticancer treatment for patients with advanced cancer, particularly when symptoms, quality-of-life concerns or psychosocial distress are insufficiently addressed [55]. In the ICU, early palliative care involvement may be especially useful in the presence of advanced disease, repeated hospitalizations, high symptom burden, frailty, poor performance status, lack of further oncological options or uncertainty about the proportionality of organ support. It can also help clarify the patient’s values and preferences before loss of decision-making capacity, which is common during critical illness because of delirium, sedation, mechanical ventilation or neurological deterioration [30,31,54].

Importantly, palliative care should not be presented as the opposite of intensive care. In many patients, both approaches may coexist: ICU support may be offered for a reversible complication while palliative care addresses symptoms, communication and goals of care. This is especially relevant when a time-limited ICU trial is proposed, as predefined reassessment points and clear communication can reduce conflict and avoid prolonged non-beneficial treatment [31,32,54].

When ICU treatment is unlikely to achieve meaningful recovery, palliative care can support transition to comfort-focused care, including withdrawal or withholding of life-sustaining therapies, symptom relief and family support. The goal is to ensure that treatment intensity remains aligned with medical realism and with what matters most to the patient.

## 12. Ethical Considerations in Treating Patients with Cancer in the ICU

Ethical conflicts are frequent throughout the course of oncological disease and may become particularly relevant when ICU admission or escalation of organ support is considered. In this context, clinical decisions should aim to maximize benefit, minimize harm and relieve suffering, while respecting the patient’s values, preferences and goals of care.

Individualizing interventions requires careful deliberation about expected benefit, risks, proportionality and patient preferences. This approach can help avoid excessive diagnostic or therapeutic measures, support patient autonomy and promote care that remains aligned with the patient’s clinical situation and personal priorities. Compassion, prudence and clear communication are essential, particularly when discussing uncertainty, poor prognosis or possible limitations of life-sustaining treatment.

Advance care planning is especially important when patients are at risk of acute deterioration, relapse or disease progression. Whenever possible, preferences regarding ICU admission, mechanical ventilation, resuscitation, treatment limitations and surrogate decision-making should be discussed before an emergency occurs and documented clearly.

Close collaboration among oncology, intensive care, palliative care, nursing teams and other professionals is necessary to ensure a comprehensive view of the patient. Ethical care also requires awareness of proportionality, futility and distributive justice, particularly in a setting where highly complex interventions and limited resources must be used responsibly.

## 13. Proposed Algorithm

**Figure 1:**
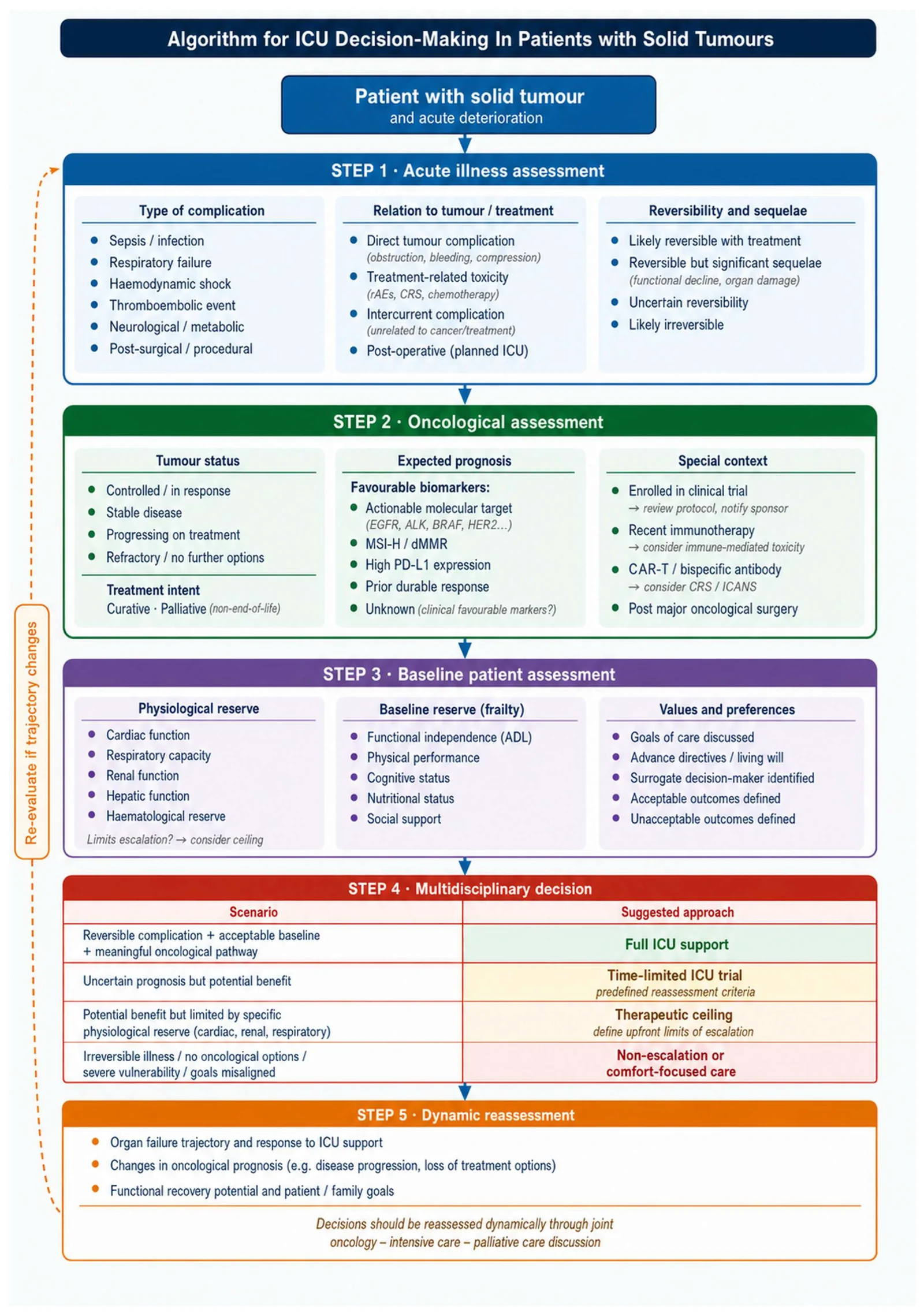
Proposed algorithm for ICU decision-making in patients with solid tumours. Initial assessment should integrate acute illness severity and reversibility, baseline functional status and frailty, cancer trajectory, available oncological options and patient preferences.

To make the algorithm operational, “acceptable baseline” refers to functional independence or a level of function that the patient considers acceptable before the acute event, interpreted alongside performance status, frailty and comorbidity rather than as a fixed score. “Potential benefit” means that organ support has a plausible path to reversing the acute complication and to recovery compatible with the patient’s goals. “Likely reversible” requires a treatable precipitating process and evidence that it can respond within a clinically meaningful interval. “Severely limited physiological reserve” denotes marked baseline frailty, major dependence, advanced irreversible comorbidity or a combination of these factors that makes recovery to an acceptable state unlikely. These descriptors should support, not replace, multidisciplinary clinical judgement (Figure 1).

According to this assessment, patients may be considered for full ICU support, a time-limited ICU trial with predefined reassessment criteria, or non-escalation/comfort-focused care. Decisions should be reassessed dynamically through joint oncology–intensive care discussion.

## 14. Challenges and Future

Improved oncological prognosis has expanded a previously under-recognized field: the care of the critically ill oncological patient. These patients may present with cancer-specific acute complications, as well as treatment-related toxicities that continue to evolve with new anticancer therapies, including immune checkpoint inhibitors and cellular therapies. In addition, they frequently develop common acute complications such as sepsis, pulmonary embolism or gastrointestinal bleeding, for which evidence on optimal management in patients with cancer remains limited. 

Addressing the expected increase in critically ill patients with cancer will require new organizational strategies. These may include telehealth-based assessment and specialized nursing triage, outpatient oncology acute care units to avoid prolonged emergency department stays, direct admission pathways, and dedicated interdisciplinary teams for oncological patients in emergency and ICU settings. Earlier outpatient decision-making about treatment intensity, based on baseline status, treatment options, age, comorbidities and patient preferences, may also help guide care before acute deterioration occurs. 

Future progress will also depend on better data and new technologies. Digital tools, national and international registries, predictive models, adaptive clinical trials for acute oncological patients, and artificial intelligence-based decision-support systems may help characterize this population, optimize workflows and improve early detection of complications. These innovations should support, but not replace, multidisciplinary clinical judgement and patient-centred decision-making.

The core principles of ICU decision-making, including assessment of reversibility, baseline function, patient preferences and the availability of effective disease-directed therapy, also apply to hematological malignancies. However, management cannot be directly extrapolated from solid tumours. Patients with hematological disorders more often have prolonged cytopenias and immunosuppression, treatment-related marrow failure, invasive fungal infection, disease-specific emergencies and, in selected cases, complications after stem-cell transplantation. These factors may alter both the immediate differential diagnosis and the anticipated trajectory of organ failure, and they require early input from a hematology team. A dedicated discussion of this population is beyond the scope of the present solid-tumour review.

## 15. Conclusions

Critically ill patients with solid tumours require individualized, multidisciplinary assessment rather than exclusion based on cancer diagnosis, metastatic status or treatment intent. ICU decisions should integrate acute illness severity and reversibility, organ failures, baseline function and frailty, cancer trajectory, available treatments, expected recovery and patient preferences.

Early collaboration between oncology and intensive care can prevent both inappropriate escalation and premature exclusion from potentially beneficial support. When prognosis is uncertain, a time-limited ICU trial with predefined reassessment criteria may provide a balanced approach. Palliative care, advance care planning and consistent communication should be integrated early. Future research should evaluate structured pathways, better prognostic tools and outcomes beyond survival, including function, quality of life and the ability to resume meaningful cancer treatment.

## Figures and Tables

**Table 1:** Clinical factors associated with more or less favourable outcomes in patients with solid tumours considered for ICU admission.

Clinical Factor	Generally More Favourable	Generally Less Favourable
**Type of ICU admission**	Planned postoperative monitoring or early ICU assessment	Unplanned admission after clinical deterioration
**Timing of ICU referral**	Early referral before multiple organ failure	Delayed referral after organ failure progression
**Acute illness**	Single, potentially reversible complication	Refractory shock, uncontrolled sepsis or multiple organ failure
**Baseline status**	ECOG 0–1, functionally independent	Poor PS, frailty, dependency or cognitive impairment
**Cancer trajectory**	Controlled, responding or slowly progressive disease	Rapid progression or uncontrolled tumour burden
**Treatment context**	Effective oncological options available	No realistic oncological treatment options
**Treatment-related complication**	Potentially reversible toxicity with specific management	Irreversible complication or no effective rescue strategy
**Expected recovery**	Likely return to acceptable function or treatment pathway	Severe expected dependency or inability to resume meaningful care

ECOG/PS: Eastern Cooperative Oncology Group Performance Status, ICU: Intensive Care Unit.

## Data Availability

No new data were created or analyzed in this study. Data sharing is not applicable to this article.
